# Promise and Implementation of Proteomic Prostate Cancer Biomarkers

**DOI:** 10.3390/diagnostics8030057

**Published:** 2018-08-29

**Authors:** Agnieszka Latosinska, Maria Frantzi, Axel S. Merseburger, Harald Mischak

**Affiliations:** 1 Mosaiques Diagnostics GmbH, 30659 Hannover, Germany; latosinska@mosaiques-diagnostics.com (A.L.); frantzi@mosaiques-diagnostics.com (M.F.); 2 Department of Urology, University Clinic of Schleswig-Holstein, Campus Lübeck, 23562 Lübeck, Germany; Axel.Merseburger@uksh.de

**Keywords:** active surveillance, biomarkers, diagnosis, Prostate Cancer, proteomics

## Abstract

Prostate cancer is one of the most commonly diagnosed malignancy and the fifth leading cause of cancer mortality in men. Despite the broad use of prostate-specific antigen test that resulted in an increase in number of diagnosed cases, disease management needs to be improved. Proteomic biomarkers alone and or in combination with clinical and pathological risk calculators are expected to improve on decreasing the unnecessary biopsies, stratify low risk patients, and predict response to treatment. To this end, significant efforts have been undertaken to identify novel biomarkers that can accurately discriminate between indolent and aggressive cancer forms and indicate those men at high risk for developing prostate cancer that require immediate treatment. In the era of “big data” and “personalized medicine” proteomics-based biomarkers hold great promise to provide clinically applicable tools, as proteins regulate all biological functions, and integrate genomic information with the environmental impact. In this review article, we aim to provide a critical assessment of the current proteomics-based biomarkers for prostate cancer and their actual clinical applicability. For that purpose, a systematic review of the literature published within the last 10 years was performed using the Web of Science Database. We specifically discuss the potential and prospects of use for diagnostic, prognostic and predictive proteomics-based biomarkers, including both body fluid- and tissue-based markers.

## 1. Introduction

One million men are diagnosed with Prostate Cancer (PC) worldwide and over 300,000 are dying annually of the disease [1]. This corresponds to more than 3000 newly diagnosed cases and around 841 deaths every day. Based on the available incidence and mortality data and when considering male population only, PC is defined as the second most commonly diagnosed cancer and fifth leading cause of mortality [1].

**Figure 1:**
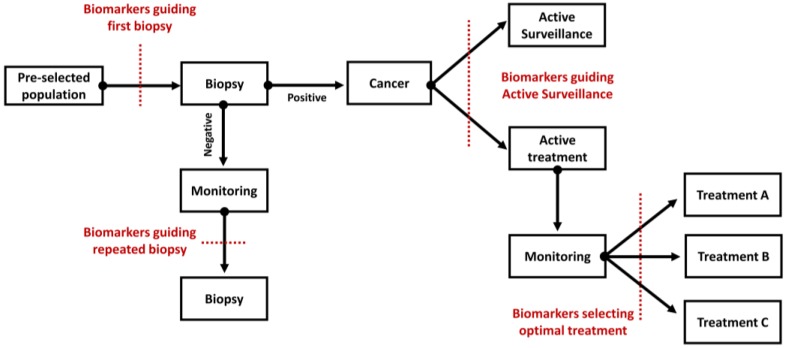
Schematic outline presenting the key elements of routine clinical practice in the management of patients with prostate cancer. The context of use for novel biomarkers is indicated in each case.

On a global scale, the rate of PC incidence varies more than 25-fold, with highest age-standardized rates in Australia/New Zealand, North America, as well as Western and Northern Europe [1]. This is likely a consequence of the frequent Prostate Specific Antigen (PSA) testing. However, among diagnosed cancer, around 45% present with an indolent course (Gleason Score (GS) < 7, PSA < 10 ng/mL) [2], which is unlikely to progress in the absence of curative treatment. As such, there is a burden related to overtreatment, along with psychological burden like growing anxiety. While PSA is a minimally invasive test, it lacks specificity resulting in high level of false-positive results that consequently lead to unnecessary biopsies. Biopsy is an invasive procedure and is associated with several side effects (e.g., haematospermia, haematuria, rectal bleeding, prostatitis, and others) [2]. Moreover, this method relies on random sampling and is prone to errors. A high proportion of biopsy-based tumor gradings are either up- or down-scaled after radical prostatectomy (RP), contributing to over- or under-treatment. Furthermore, pathological and clinical parameters are not able to predict accurately the outcome of the disease. Even though several improvements have been made, such as the introduction of multiparametric magnetic resonance imaging guided biopsy [3], the discrimination between indolent and aggressive cancer is still problematic, resulting in inaccurate risk stratification [3]. In addition, when cancer becomes metastatic the prognosis is poor. The median survival for patients with metastatic castration resistance PC (mCRPC) is 24 months. Consequently, while patients diagnosed with indolent disease are at risk of being overtreated, patients suffering from aggressive disease lack effective therapeutic options. The main clinical questions that remain to be answered, as depicted in Figure 1, are the following:

1. Which patient needs a first or a repeated biopsy?
2. Which patient needs immediate treatment?
3. Who is likely to benefit from the indicated treatment?

To address these points and improve the current care for patients with PC, several tests have become commercially available: (1) tests approved by U.S. Food and Drug Administration (FDA) and (2) tests available via Clinical Laboratory Improvement Amendments (CLIA)—certified laboratories [4,5]. The first group consists of PSA, Prostate Health Index, and Prostate Cancer Gene 3 (PCA3); while the second group includes, but it is not limited to (1) gene-based tests (e.g., SelectMDx, ExoDx, Mi-Prostate score, ConfirmMDx, Prostate Core Mitomic test, OncotypeDX, Prolaris, Decipher, and AR-V7), (2) protein-based tests (e.g., 4Kscore Test and ProMark) and (3) metabolic-based tests (e.g., Prostarix) (Table 1). Most of these commercially available tests measure genomic markers, as a result of advanced maturity and accessibility of the gene-based technologies. Although progress has been made, most of these tests have not been fully integrated into clinical practice and further prospective studies are recommended to validate their clinical utility and cost-effectiveness [5].

In parallel to gene-based approaches, extensive research has been conducted to develop protein-based tests, with some of them being also commercially available. Proteins are responsible for the regulation of biological functions and their investigation at the global level becomes possible due the advancements in Mass Spectrometry (MS)-based technologies. The proteome, which is defined as a complete set of proteins in cell, tissue, or organism that are expressed at a certain point in time, has a highly dynamic nature and contains information on molecular disease determinants. Thus, the investigation of the individual proteome and identification of proteins associated with disease enable to identify biomarkers. For that reason, proteomics approaches have been broadly applied to investigate diagnostic, prognostic, and predictive biomarkers.

The main aim of this review is to provide an overview on the currently available proteomics biomarkers that were developed to address the clinical needs for PC, emphasizing their clinical implementation. As proteomics-derived markers have still not been routinely applied in clinical practice, challenges, and some potential solutions, to further support clinical implementation are discussed in the outlook.

## 2. Literature Search and Review Strategy

**Figure 2:**
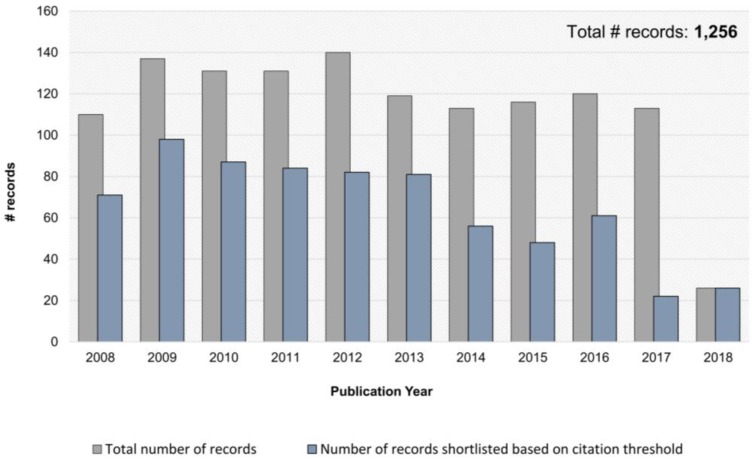
Overview of the number of articles published over last 10 years covering the TOPIC: (biomarker* or marker*) AND TOPIC: (proteome*) AND TOPIC: (“prostate cancer” or “prostate adeno*”), as retrieved using Web of Science.

**Figure 3:**
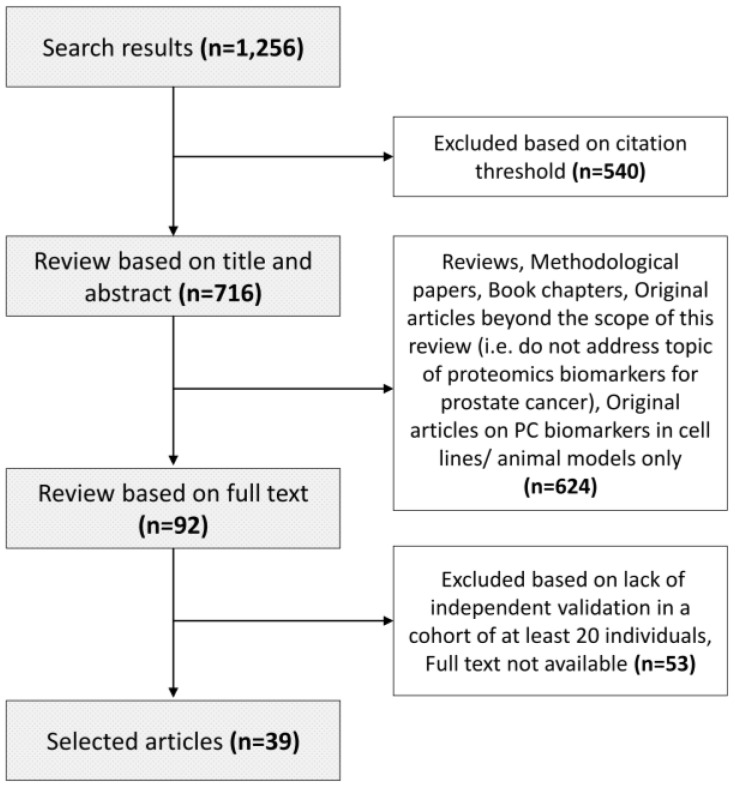
Graphical representation of the conducted literature search and review strategy.

A systematic literature search was performed through the Web of Science platform on 6 June 2018. Records were retrieved from all databases based on the following search criteria: (1) TOPIC: (biomarker* or marker*) AND TOPIC: (proteome*) AND TOPIC: (“prostate cancer” or “prostate adeno*”) and (2) Timespan: 2008-2018. The search resulted in retrieval of 1256 manuscripts, as presented in Table S1. The manuscripts were further shortlisted based on the number of citations. Only manuscripts published between 2008 and 2015 with at least 10 citations were considered; whereas between 2016 and 2018 a lower threshold was applied as follows: at least five citations for 2016, at least four citations for 2017, and no citation threshold for 2018 (Figure 2). These 716 manuscripts were subsequently screened based on title and abstract for their relevance in the field of proteomics biomarkers in prostate cancer. Methodological papers, reviews, editorials, commentaries, and manuscripts performed only in cell lines/animal models were excluded, leading to the list of 92 papers. For the latter, the full text was screened, and only original manuscripts presenting biomarkers for prostate cancer that were validated in the independent cohort (n ≥ 20) were selected. Collectively, 39 papers are presented in the context of this review (Table S2). A graphical representation of the search and review strategy is presented on Figure 3.

## 3. Proteomics Approaches for PC Biomarker Development

A broad range of proteomics platforms has been applied to investigate body fluid-based (urine, blood, and seminal plasma) and tissue-based biomarkers for PC. Based on the literature search and also in agreement with the general consensus in the field, biomarker discovery is typically performed using platforms that allow for global assessment of protein alterations, including both gel-based and gel-free methods; whereas validation of the selected biomarkers is based mostly on classical immuno-based assays and targeted MS. An overview on the advantages and disadvantages of proteomics approaches is provided in a past paper [33]. The most frequently applied approaches (as itemized in Table S3) are briefly described below, while more information can be found in other review articles [34,35,36,37,38,39,40,41].

### 3.1. Platforms Applied for Biomarker Discovery

Discovery of proteomics biomarkers in the context of PC was carried out using both gel-based (i.e., two-dimensional gel electrophoresis (2DE) and two-dimensional difference gel electrophoresis (2D-DIGE) in combination with MS for protein identifications) as well as gel-free approaches (liquid chromatography coupled with tandem mass spectrometry (LC-MS/MS), capillary electrophoresis coupled to mass spectrometry (CE-MS)), with LC-MS/MS being most frequently applied. Gel-based techniques, like 2DE and 2D-DIGE (modification of 2DE), involve separation of the proteins using isoelectric focusing based on their charge, followed by separation in sodium dodecyl sulfate (SDS)- polyacrylamide gel based on their mass. As a result, a two-dimensional map of proteins is generated where separated proteins appear as “spots”. The main difference between 2DE and 2D-DIGE is the use of fluorescent cyanine dyes (Cy2, Cy3, and Cy5) to label the proteins prior to isoelectric focusing. This allows for simultaneous separation of three samples, usually representing a control group, a case group, and an internal standard (pool of equal amount of control and case sample). Compared to classical 2DE, 2D-DIGE decreases gel-to-gel variability and improves quantification and mapping of spots across different gels. However, neither 2DE nor 2D-DIGE provide a direct link to protein identity. For that purpose, spots of interest are excised from the gel, proteins are then digested (in-gel digestion) using trypsin, and the peptide extracts are analyzed by MS.

With the advancements of MS, new gel-free approaches have been developed and applied to comprehensively investigate the proteome. Particularly LC-MS/MS analysis has been most frequently applied to identify novel markers for PC. The proteins are initially digested into peptides, followed by chromatographic separation. Frequently, considering the high complexity of the protein/peptide extracts, additional steps are required to decrease the sample complexity and improve on the identification rate of low abundant proteins. This includes among others (a) depletion of most abundant proteins, (b) enrichment of the sample in the proteins or peptides of interest (e.g., glycopeptide enrichment), or (c) multidimensional separation of peptides through combined chromatographic strategies prior to MS analysis. Subsequent MS/MS analysis includes peptide ionization and separation of precursor ions based on mass over change ratio (MS1 level) and its fragmentation (MS2 level), with the latter used as a basis for determination of peptide sequences. This analysis generates data of high complexity, especially when multiple samples are analyzed in the context of a single experiment. Quantification of MS-based proteomics findings is performed by either a metabolic or a chemical labeling (label-based) or without labeling by using spectral counting or intensity-based method (label-free approaches).

Another approach, CE-MS, is based on the analysis of low molecular weight proteins and peptides (<20 kDa) that are present in body fluids. In this analysis, molecules are separated in an uncoated fused silica capillary. As in LC-MS/MS, the molecules are ionized and then analyzed by MS. Through this approach several biomarker patterns for urogenital malignancies have been already developed [42,43,44,45,46,47]. In comparison to the other platforms described above, CE-MS has been analytically validated as a routine diagnostic platform [48] and has also received a “Letter of Support” from the U.S. FDA for its application in chronic kidney disease [49]. A comparison of advantages and disadvantages of CE-MS/MS and LC-MS/MS is presented in a previous paper [50].

### 3.2. Platforms Applied for Biomarker Verification/Validation

Most of the platforms that are applied for validation are based on antibodies to detect protein biomarkers [35]. This includes assays such as Enzyme-linked immunosorbent assay (ELISA), Western Blot (WB), and Immunohistochemistry (IHC). ELISA has been mostly applied for validation of biomarkers in body fluids, IHC has been applied to determine the level of potential biomarkers in tissue samples, while WB has been used in the context of both body fluids and tissue samples. However, selectivity of these methods depends mostly on the specificity of the applied antibodies and the complexity of the matrix in which the analyte is measured. Moreover, these methods are typically limited to measure single biomarkers, while for complex and heterogeneous disease such as PC, panels comprised of multiple biomarkers have reported better performance [51]. As an alternative to classical immuno-based methods, MS-based approaches have emerged, with the focus placed on the measurement of multiple selected biomarkers within a single assay. This includes selected and multiple reaction monitoring (SRM/MRM) performed most commonly using triple quadrupole MS. As outlined in detail in several reviews [52,53,54], the peptide ions of interest are selected in the first quadrupole, followed by fragmentation in the second quadrupole, and selection of specific fragments ions for detection in the third quadrupole. When SRM is applied to investigate multiple product ions, this method is referred as MRM.

## 4. Proteomics-Based Biomarkers for Prostate Cancer

Proteomics approaches have been widely employed in PC biomarker research, reflected by the numerous studies that have been published over the last 10 years. The research focus is on the development of urinary, blood, seminal plasma, and tissue-derived biomarkers in the context of diagnosis, risk stratification, and prediction of treatment response. An overview of the types of clinical samples applied in the context of PC biomarker development, along with main advantages, disadvantages and context of use is provided in Table 2. As a result of these investigations, numerous biomarkers have been proposed for the management of PC patients. The shortlist of the most promising findings (selected based on independent validation and performance assessment in >100 samples) is presented in Table 3. In the following subsections, studies reporting on body fluids and tissue-derived biomarkers are described.

### 4.1. Body Fluids Biomarkers

Body fluids are the preferable source of noninvasive or minimally invasive biomarkers, due to the limited associated side effects from sampling. These biomarkers are promising for patients with PC, for which invasive biopsy is the current “gold standard” of clinical care.

#### 4.1.1. Urinary Biomarkers 

Due to noninvasive collection, urinary biomarkers are an ideal alternative to regular extensive biopsy procedures for diagnostic purposes and for disease monitoring. To identify urinary biomarkers for PC, both full urine and urine collected after digital rectal examination (DRE) has been analyzed. Prostate massage performed during DRE enriches the urine samples with PC-originated material such as cancer cells and prostatic fluids [61], making it a particularly attractive source for PC biomarkers. In addition, combination of individual biomarkers into panels, has been applied, resulting in increased diagnostic accuracy in comparison to single markers. An overview on studies employing urinary proteomics is provided below.

A. Biomarkers for cancer detection

Numerous urinary-based proteomics multimarker panels for cancer detection have been identified. Davalieva et al. investigated the urinary proteome from patients with PC (n = 8) and benign prostatic hyperplasia (BPH, n = 16) using 2D-DIGE in combination with matrix assisted laser desorption ionization combined with tandem time of flight MS (MALDI-TOF/TOF) [62]. Comparative analysis revealed 23 unique proteins that significantly differ between cancer and control group (14 upregulated and nine downregulated in PC). Nine of these 23 proteins were involved in acute phase response signaling pathway: Protein AMBP (AMBP), Apolipoprotein A-I, Fibrinogen alpha chain, Fibrinogen gamma chain, Haptoglobin (HP), Inter-alpha-trypsin inhibitor, Alpha-1-antitrypsin, Transferrin (TF), and Transthyretin [62]. The differential abundance of TF, AMPB, and HP was confirmed in an independent, small set of urine samples (n = 16 PC, n = 16 BPH) using immunoturbidimetry (area under the ROC curve (AUC) = 0.75 for TF; AUC = 0.74 for AMBP and AUC = 0.72 for HP). Combination of HP and AMBP increased the performance (AUC = 0.85) [62]. These results should be confirmed in a larger cohort.

Jedinak et al. applied 8-plex isobaric tags for relative and absolute quantitation (iTRAQ) labeling (n = 4 PC, n = 4 BPH) followed by multidimensional chromatographic separation and MALDI-TOF/TOF to discriminate between localized PC and BPH [56] and identified significant changes in urinary levels of 25 proteins. Based on the availability of antibodies, nine proteins were further evaluated using WB on 173 urine samples (n = 90 PC, n = 83 BPH), and significant increase of β-2-microglobulin (β2M), Pepsin A-3 (PGA3), and Mucin-3A (MUC3A) 25kDa were verified. When combining individual markers into a panel, the AUC value increased significantly to 0.71 (*p* < 0.001). Further improvement was observed after integration of PSA in the panel (AUC = 0.81, *p* < 0.001)) [56].

CE-MS was applied to investigate naturally occurring urinary peptides as plausible biomarkers to guide prostate biopsy [45,47]. In the first study, a panel comprised of 12 urinary peptides was established in a cohort of 86 individuals (n = 51 PC, n = 35 patients with negative biopsy) and its performance was further validated in the blinded prospective manner (n = 118 PC, n = 95 patients with negative biopsy) [47]. The developed panel reached AUC of 0.70 in the validation cohort. When combined with age and percent free PSA, the AUC increased to 0.82 [47]. Application of MS/MS allowed for the identification of four peptides included in the panel, which corresponded to Sodium/potassium-transporting ATPase γ, Collagen α-1 (III), Collagen α-1(I), and Psoriasis susceptibility 1 candidate gene 2 protein [47]. In a subsequent study [45], the performance of the developed model in a group of German patients with PSA value > 4 ng/mL or suspicious DRE (n = 49 PC, n = 135 patients with negative biopsy) was assessed. The panel showed significantly better diagnostic accuracy (AUC = 0.72) in comparison to total serum PSA (AUC = 0.60, *p* = 0.023). Moreover, cost-effectiveness of the proposed biomarkers to guide biopsy in patients with suspicious PSA test was supported [45].

Few exploratory studies were performed to evaluate biomarkers identified in previous investigations. Zinc-α2-glycoprotein (AZGP1) [55], Flotillin-2 and Protein/nucleic acid deglycase DJ-1 (PARK7) [63] were proposed as biomarkers for cancer diagnosis. Katafigiotis et al. analyzed the urinary levels of AZGP1 in a cohort of 127 consecutive candidates for a transrectal ultrasound prostatic biopsy using WB [55]. Combination of AZGP1 with PSA significantly improved diagnostic accuracy (AUC = 0.75, *p* = 0.010), compared to PSA only (AUC = 0.65) [55]. Interestingly, the urinary levels of AZGP1 were higher in patients with PC compared to patients with negative biopsy, while the opposite trend was observed at the tissue level: strong expression in benign epithelium and weak/negative expression in higher GS malignant tissue [55].

Wang et al. measured selected urinary exosomal proteins using immuno-based assays (WB and ELISA) [63]. Based on the WB measurements (n = 16 PC, n = 16 healthy donors), receiver operating characteristic curve (ROC) analysis for Flotillin-2 showed an AUC value of 0.91; while ELISA analysis of flotillin-2 (n = 19 PC, n = 15 healthy donors) resulted in an AUC of 0.65. The urinary exosomal level of PARK7 was also assessed by ELISA resulting in an AUC of 0.71. However, these results should be further evaluated in a larger cohort including disease-matched controls, rather than healthy individuals.

Sequeiris et al. applied SRM to validate 64 proteins, retrieved from in-house proteomics investigations and literature [64,65,66,67], in urinary extracellular vesicles (EVs) using a cohort of 107 individuals (n = 53 PC, n = 54 patients without PC e.g., BPH, inflammation, and high-grade prostatic intraepithelial neoplasia (HGPIN)) [57]. The differential abundance of 14 out of 64 proteins was confirmed between PC and control group [57]. The diagnostic performance was assessed for individual proteins and their combinations, with the best performance observed, when combining Transglutaminase-4 (TGM4) and Adseverin (ADSV) (AUC = 0.65) [57]. Further validation of these proteins using IHC (n = 136 PC tissues, n = 98 benign prostatic tissues) supported their differential expression, in agreement with urinary data [57]. ROC analysis based on the tissue data supported their diagnostic potential (AUC of 0.81 for TGM4 and an AUC of 0.73 for ADSV) [57].

The proteome of urinary EVs was investigated by Fujita et al. [68] using iTRAQ and LC-MS/MS in six patients with negative biopsy, six PC patients with GS = 6, and six PC patients with GS = 8–9. An increase of 11 proteins in patients with PC vs. patients with negative biopsy (ratio >1.5, *p* < 0.05) was observed [68]. These proteins were subsequently validated in an independent set of 29 urine samples using SRM/MRM, confirming only one protein, Fatty acid-binding protein 5 (FABP5) (*p* = 0.009) [68]. FABP5 levels in urinary EVs were associated with GS (*p* = 0.011). Initial ROC analysis resulted in an AUC of 0.76 (*p* = 0.027) for detection of PC as well as an AUC of 0.86 (*p* = 0.002, n = 26) for detection of PC with a GS ≥ 7 [68]. Increased expression of FABP5 was also found in tissue and serum of patients with metastatic vs. localized PC [69], in line with the data on EVs. This protein warrants further validation in larger cohort, in a clearly defined clinical context.

Casanova-Salas et al. identified Aldehyde dehydrogenase 1A3 (ALDH1A3) as a potential PC biomarker by investigation of miR-187 targets using 2D-DIGE analysis of PC-3 cell lines transfected with a miR-187 precursor and miRNA mimicking negative controls [70]. Analysis of ALDH1A3 expression in tissue samples (n = 203) revealed a significant increase in PC [70]. However, assessment of diagnostic potential of urinary ALDH1A3 in patients with positive (n = 63) and negative prostate biopsy (n = 60) did not support its role as a useful biomarker: with an AUC of 0.59 (*p* = 0.083) it was inferior to PSA (AUC = 0.61, *p* = 0.036) [70].

B. Biomarkers for risk stratification to guide therapeutic intervention.

Only few urine-based proteomics studies reported on the identification of biomarkers for risk stratification. Similarly, to the CE-MS studies for cancer detection (as outlined above), naturally occurring urinary peptides were investigated for their potential to detect clinically significant PC. A CE-MS based panel, consisting of 19 peptide markers was established in a cohort of 823 PC patients with low PSA levels (<15 ng/mL). Validation in an independent test set of 280 PC patients resulted in an AUC value of 0.79. Combination with age and the European Randomized study of Screening for Prostate Cancer (ERSPC) risk calculator resulted in an AUC value of 0.81, significantly higher compared to the ERSPC alone (AUC = 0.71; *p* = 0.0375) (manuscript submitted).

In the study by Sequeiris et al. [57] (as summarized above) the proteome of urinary EVs was investigated for the value of previously identified potential biomarkers [64,65,66,67] to discriminate between low and high grade PC. SRM analysis revealed that 45 out of the 64 proteins were significantly altered between low-grade (n = 22, GS ≤ 3 + 4) and high grade PC (n = 31) [57], with the best performance obtained for the combination of 5 proteins (Prostatic acid phosphatase, PSA, CD63 antigen (CD63), N-sulphoglucosamine sulphohydrolase, and Putative glycerol kinase 5) reaching an AUC of 0.70 [57]. Further analysis using IHC (50 low grade PC, 86 high grade PC) indicated significant alteration only for CD63 [57].

To assess if body fluids contains a tissue glycopeptide signature, N-linked glycopeptides from urine and serum were investigated using LC-MS/MS [71], while data on tissue glycopeptides were retrieved from the literature. Approximately 40% of the glycopeptides originating from PC tissues were also found in urine; the overlap with serum was lower (13%) [71]. Along these lines, tissue-derived differentially expressed glycoproteins associated with aggressive PC were compared with acquired urine and serum profiles; while most of the tissue proteins were detected in urine, but not in serum. Based on these results, urinary glycoproteomics profiles were acquired from additional 20 samples from patients with low-grade (n = 10) and high-grade PC (n = 10) [71]. A significant decrease of Inactive tyrosine-protein kinase 7 (PTK7), ICOS ligand, AZGP1, Fibrillin-1, and Golgi apparatus protein 1 was reported in urine from patients with high grade PC [71]. Further investigation of the potential value of these markers to discriminate between aggressive and nonaggressive PC needs to be planned.

#### 4.1.2. Blood-based Biomarkers

Due to minimally invasive sampling, blood-based (mostly serum) proteomics biomarkers for PC have been extensively investigated, mostly aiming at diagnostic purposes. However, proteomic analysis of blood is challenging, due to its high complexity and the broad range of protein concentration. As such, decreasing of the samples’ complexity by the application of depletion or enrichment strategies or multidimensional chromatographic separation of peptides prior MS has been applied. The most promising blood-based biomarker studies are presented in the following subsections.

A. Biomarkers for detection of cancer

Larkin et al. applied iTRAQ labeling in combination with multidimensional LC-MS/MS to investigate serum proteome in patients with T1-T2 PC (n = 20), T3-T4 PC (n = 20), benign disease (n = 15), and healthy controls (n = 20) [72]. The samples were pooled for each group and labeled with two different tags. Forty differentially regulated proteins with consistent regulation trends were identified. From those seven proteins (Delta-sarcoglycan, Pre-rRNA-processing protein TSR1 homologue (TSR1), PSA, von Willebrand factor A domain containing protein 5B2, Serum amyloid A protein (SAA1), Proto-oncogene tyrosineprotein kinase Src, and Cystatin-C) were selected for validation based on their discriminatory capability between the analyzed groups and the antibody availability. ELISA analysis (n = 20 T1–T2, n = 20 T3–T4, n = 20 BPH, n = 20 healthy controls) confirmed differential expression of SAA1 and PSA across groups (pairwise analysis). In addition, TSR1 was found consistently upregulated across individual groups when comparing to healthy, with significant change reported only for T1–T2 vs. healthy controls (*p* = 0.013). ROC analysis for individual markers showed an AUC of 0.68 for PSA (*p* = 0.006), 0.60 for SAA1 (*p* = 0.117), and 0.61 for TSR1 (*p* = 0.081) in discriminating nonmalignant (BPH + healthy controls) from malignant samples. Combination of TSR1 and PSA improved the diagnostic performance (AUC = 0.73) [72].

In a study by Byrne et al. immuno-depleted serum samples from 12 PC patients undergoing RP (n = 6 GS5, n = 6 GS7) were analyzed using 2D-DIGE followed by LC-MS/MS [73]. Among the 14 proteins identified as potential biomarkers, differential expression of AZGP1 (upregulated in GS7 vs. GS5) and Pigment epithelium-derived factor (PEDF, downregulated in GS7) was validated in serum (n = 18 PC GS5, n = 19 PC GS7, n = 13 BPH) and tissue samples (n = 29 for PEDF, n = 27 for AZGP1) [73]. The highest diagnostic accuracy for discrimination between cancer vs. BPH was obtained when combining AZGP1, PEDF, and PSA (AUC = 0.85) [73].

Burgess et al. identified Heat shock protein 90 alpha (HSP90α) through the investigation of proteins associated with serum Alpha-2 macroglobulin using immunoaffinity enrichment and LC-MS/MS (six patients with androgen independent, metastatic PC and six patients with no evidence of cancer) [74]. HSP90α was significantly upregulated in sera from cancer patients and exhibited an AUC of 0.83 for discrimination between cancer (n = 18) and control patients (n = 13) [74].

Potential biomarkers were also identified using cell lines or animal models, followed by validation in clinical specimens. Sardana et al. analyzed conditioned media from PC cell lines (PC3-bone metastasis, LNCaP-lymph node metastasis, and 22Rv1-localized to prostate) using two-dimensional LC-MS/MS [75]. Further evaluation of biological function, tissue specificity, as well as assessment of the overlap compared to previous investigations of serum and seminal plasma, resulted in the selection of four proteins [Follistatin, Chemokine (C-X-C motif) ligand 16, Pentraxin 3 and Spondin 2 (SPON2)] to be validated in 42 serum samples from patients with or without PC [75]. Serum levels of these proteins were higher in patients with PC, but the performance of these potential biomarkers has to be assessed in detail [75].

One of these proteins, SPON2, was also highlighted in a study by Qian et al. [76], investigating conditioned media from BPH-1 (BPH epithelial cell line), LNCaP, and C4-2 (androgen independent derivative of LNCaP) cell lines using 2DE in combination with LC-MS/MS [76]. SPON2 was found to be expressed only in the media of androgen receptor (AR)-positive PC cell lines. Subsequent analysis of clinical samples showed that the level of SPON2 is significantly higher in patients with PC than healthy controls, both at the level of tissue (n = 44 PC, n = 19 BPH, n = 10 normal prostate tissue) and serum (n = 70 PC, n = 13 healthy donors). ROC analysis based on measurements of SPON2 in serum showed very good discrimination between cancer and healthy (AUC = 0.94) [76]. Even though the diagnostic performance was high, the use of healthy individuals as controls may not be appropriate when investigating PC biomarkers. As such, further investigation of diagnostic performance of this protein is warranted.

Exosomes were isolated from the conditioned media of androgen-dependent (LNCaP) and androgen-independent (C4, C4–2, and C4–2B; sublines of LNCaP) cell lines [77]. The exosomal proteome was analyzed using iTRAQ and MALDI-TOF/TOF, resulting in identification of a total of 153 proteins. Based on the >1.5-fold change, eight proteins were defined as elevated in exosomes of C4–2B compared to LNCaP cells. Gamma glutamyltransferase 1 (GGT1) was found to be upregulated in both C4–2 and C4–2B cells. Considering the biological relevance of GGT1 in other cancer types, the activity of exosomal GGT1 was assessed in more detail in serum (n = 31 PC and n = 8 BPH). Significantly higher activity was detected in PC vs BPH. This finding was further supported through the analysis of tissue samples (n = 50 PC, n = 50 BPH) [77]. Based on the measurement of GGT1 activity, ROC analysis was performed, resulting in an AUC of 0.71 [77].

Cima et al. analyzed N-linked glycoproteins in serum and tissue samples from wild type and phosphatase and tensin homolog (PTEN)-null mice using LC-MS/MS [58]. A total of 126 proteins were shortlisted for evaluation in human samples (serum and tissue) considering the following criteria: significance, detectability in serum, as well as prostate specificity. As a result of this study three biomarker panels were developed using regression models. This included serum signatures for: (1) stratification based on PTEN status (Thrombospondin-1, Metalloproteinase inhibitor 1, Complement factor H, and Prolow-density lipoprotein receptor-related protein 1), (2) PC diagnosis (Hypoxia upregulated protein 1, Asporin, Cathepsin D, and Olfactomedin-4), and (3) discrimination of GS <7 or ≥7 (polypeptide GalNAc transferase-like protein 4, Fibronectin, AZGP1, Biglycan, and Extracellular matrix protein). Performance of the developed signatures was as follows. (1) PTEN signature: AUC = 0.82 (n = 54), (2) diagnostic signature: AUC = 0.73 (n = 105), and (3) Gleason signature: AUC = 0.79 (n = 54) [58]. Combination of the diagnostic signature with PSA increased the performance, reporting an AUC of 0.84. The above signature was also initially validated in a small, independent cohort of 37 patients [58]. However, the assessment of validity and value of the biomarker panels in an independent and well-powered prospective investigation is yet missing.

Parallel proteomics and transcriptomics analysis of a very small set of tissue samples (n = 4 cancer tissue and four adjacent normal tissues) was also applied to discover serological biomarkers for PC [78]. Cross-correlation of -omics data revealed 14 potential biomarkers to be differentially expressed at both mRNA and protein level. Validation in 140 serum samples (n = 84 PC, n = 35 BPH, n = 21 healthy donors) [78] demonstrated a significant increase of Methylcrotonoyl-CoA carboxylase beta chain, mitochondrial, Tumor necrosis factor receptor-associated protein 1, and Inosine monophosphate dehydrogenase II (IMPDH2) in cancer patients when comparing to BPH or healthy controls. In addition, significantly higher levels of serum IMPDH2 were detected in cancer patients with GS ≥ 8 vs. GS < 8, and also associated with metastasis [78].

Ueda et al. analyzed the low-molecular weight plasma proteome using LC-MS/MS [79]. Analysis of 116 plasma samples (n = 73 PC, n = 19 BPH, n = 24 healthy donors) revealed 189 cancer specific peptides [79]. Among those, Neuropeptide-Y (NPY) was identified and validated using MRM in independent set of plasma samples (n = 110). Gradual increase of protein abundance was observed from healthy (n = 26), BPH (n = 19) and across cancer cases (n = 65) stratified by GS. However, NPY (AUC = 0.72) does not outperform PSA (AUC = 0.82) when comparing PC vs. control group (BPH and healthy donors). Combination of these two markers resulted in AUC of 0.88 [79]. NPY improved on specificity of PSA, without significant decrease of its sensitivity [79].

B. Biomarkers for risk stratification to guide therapeutic intervention.

Proteomics approaches have also been applied to identify biomarkers for risk stratification. Rehman et al. applied a 4-plex iTRAQ-based quantification methodology to assess pooled serum samples from patients with localized PC (incl. 10 patients defined as ‘nonprogressing’ and 10 ‘progressing’ based on the change of PSA level over the 5 years of monitoring), metastatic PC (n = 5), and BPH (n = 5) [80]. 22, 19, and 35 differentially expressed proteins were identified for “nonprogressing”, “progressing”, and metastatic PC when comparing to BPH, respectively. Assessment of the changes of protein levels across disease progression indicated significant increase of Eukaryotic translation elongation factor 1 alpha 1 (eEF1A1) in “nonprogressing” PC vs. BPH, followed by a further increase along disease progression and metastasis in PC [80]. IHC analysis of tissue samples from patients with BPH and organ confined PC, and bone from patients with and without metastatic PC was performed and differential expression of eEF1A1 in organ-confined and metastatic PC was confirmed. Higher levels of eEF1A1 were observed in the osteoblasts adjacent to metastatic PC cells (n = 6) vs. osteoblasts in normal control bone samples (n = 15) [80]. However, the extremely low number of subjects in the study does not allow for solid conclusions regarding the application of eEF1A1 as biomarker.

Claudin 3 was identified as a potential biomarker through proteome analysis of exosomes extracted from conditioned media of PC3 (malignant) and PNT1A (benign) cell lines [81]. Plasma levels of Claudin 3 were subsequently determined using ELISA (n = 58 localized PC, n = 11 metastatic PC, n = 15 BPH, n = 15 healthy individuals), with increased level detected in GS ≥ 8 compared to BPH and GS ≥ 8 vs. GS6-7. When assessing the performance of Claudin 3 in patients with localized PC (GS6-7 and GS ≥ 8), AUC of 0.71 was reported [81]. However, considering the low number of patients with localized PC that were used to assess the discriminatory capability of Claudin 3, further studies are required to support this finding.

C. Biomarkers for prediction of treatment response

There is an emerging need to identify biomarkers to predict treatment response for patients with advanced PC, especially those with CRPC. To address this need, some studies have been published. However, when comparing with the development of diagnostic or risk stratification biomarkers, the results are rather limited.

In the study by Zhao et al., proteome analysis was applied to identify biomarkers able to predict response to docetaxel [82]. The initial discovery was based on comparative analysis of Docetaxel-sensitive (PC3) and -resistant (PC3-Rx) cells and resulted in identification of 85 differentially expressed proteins (fold > 1.5). Among the latter, seven proteins were defined as secreted, and those with the highest fold change (Macrophage inhibitory cytokine 1 (MIC-1)—top upregulated; Anterior gradient 2 homologue—top downregulated) were evaluated in serum samples. Increased levels of MIC-1 after cycle one of chemotherapy (n = 28 Docetaxel/PI-88, n = 8 Docetaxel alone, n = 2 Mitoxantrone) were associated with cancer progression (*p* = 0.006) and shorter survival after treatment (*p* = 0.002) [82]. Further validation of these findings in larger cohorts is required.

Serum biomarkers predictive of treatment response to everolimus in patients with mCRPC were assessed as part of a phase 2 clinical trial [83]. A total of 37 chemotherapy naïve patients were enrolled, including 13 patients reaching primary endpoint (progression free survival at 12 weeks after treatment-absence of PSA or radiographic or clinical progression) [83]. SRM analysis was applied to investigate the levels of 40 previously identified PI3K/Akt pathway-related glycopeptides in serum samples from 28 patients [83]. Univariate analysis revealed significant association of 13 proteins with the primary end point. Among those, Carboxypeptidase M and Apolipoprotein B were found to be most strongly predictive of the outcome, with AUC of 0.85 and 0.79, respectively [83]. In addition, eight biomarkers were found to be significantly associated with progression-free survival using univariate Cox regression analysis, including four being predictive of the primary endpoint (Apolipoprotein b, Complement factor H, Ceruloplasmin, and Carboxypeptidadse M) [83].

#### 4.1.3. Seminal Plasma Biomarkers

Seminal plasma is a body fluid that consists of secretions from seminal vesicles (~65% of semen volume), prostate (~25%), testis, and epididymis (~10%) [84]. Seminal plasma resembles in its complexity the plasma proteome, with the top 10 most abundant proteins contributing to >80% of the total protein amount [84]. This represents a challenge for proteomics analysis and as such additional steps (such as depletion and prefractionation) are required to better identify low abundant biomarkers. Considering the high proximity to prostate tumor and minimally invasive collection, seminal plasma emerged as promising source of biomarkers for PC. Seminal plasma-based tests have not been frequently applied in clinical practice. This is to some extent associated with seminal plasma sampling method, which is not easily applicable and generally acceptable [84]. Moreover, collection of seminal plasma can be problematic for elderly men, as shown in the study by Neuhaus et al. (described in more details below). Only 30–50% of patients prior to RP were able to donate ejaculate [44]. This depicts well the scale of the problem, not only for the routine application in clinical practice, but also for conducting well-powered studies to develop PC biomarkers. On the other hand, seminal plasma-based tests to identify aggressive PC are preferable over invasive biopsy [84]. To be implemented in clinical practice, seminal plasma-based tests should outperform or substantially complement other noninvasive solutions available and provide diagnostic information that is not available using other methods. Studies reporting on seminal-plasma based biomarkers are presented below. In most cases, proteomics biomarkers have been developed to support discrimination between aggressive and nonaggressive PC.

A. Biomarkers for risk stratification to guide therapeutic intervention

Neuhaus et al., analyzed seminal plasma using CE-MS [44]. A panel of 11 peptide markers was developed to discriminate patients with localized (GS7 and organ-confined (<pT3a)) from advanced PC (GS7, ≥ pT3a). The initial discovery cohort included 21 PC with GS < 7 and 16 PC with GS > 7 [44]. Validation in a cohort of 33 PC patients indicated an AUC of 0.83 (*p* = 0.0055) to discriminate between localized (n = 28) and advanced disease (n = 5) [44]. While these results are highly promising, further studies were not conducted due to the lack of funding. The main criticism for not financially supporting such studies is that patients would not be willing to donate seminal plasma.

In a study by Saraon et al., a biomarker discovery study was performed using 2D LC-MS/MS on conditioned media from panel of androgen dependent, androgen independent and normal prostate epithelial cell lines [85]. Differential expression analysis was conducted to identify proteins indicative of androgen-independent and aggressive PC. Protein S (PROS1) was found only in conditioned media from all androgen-independent cell lines, thus was selected for further validation in clinical samples (tissue and seminal plasma) [85]. Elevated expression of PROS1 was shown in localized, high grade PC, both at the tissue (IHC analysis: n = 8 normal prostatic tissue, n = 40 localized PC incl. high and low grade PC) and seminal plasma level (ELISA: n = 8 patients with negative biopsy, n = 8 prostatitis, n = 8 low grade PC, n = 13 intermediate/high grade PC, *p* < 0.05) [85]. Moreover, significant increase in PROS1 expression was also observed in CRPC metastases including lung and liver (n = 19, *p* = 0.0009), lymph node (n = 28, *p* = 0.0026), and bone (n = 72, *p* = 0.0022) metastatic lesions in comparison to normal prostate samples (n = 8). ROC analysis based on the seminal plasma levels of PROS1 showed an AUC of 0.88 (*p* < 0.001) when discriminating between benign (negative biopsy, prostatitis)/low grade PC and intermediate/high grade PC [85].

B. Biomarkers for cancer detection

In parallel, development of diagnostic markers to improve detection of cancer was attempted. For that purpose, as a part of a study by Neuhaus et al., two biomarker panels were established in a training set (n = 22 PC, n = 14 chronic prostatitis, n = 9 BPH, n = 5 healthy controls) [44]. This included (1) a biomarker panel comprised of 21 seminal plasma peptides to differentiate PC and BPH from prostatitis and healthy individuals, (2) a biomarker panel of 5 peptides to discriminate between PC and BPH [44]. Based on peptide sequencing data, fragments of Semenoglein-1, Semenoglein-2, Prostatic acid phosphatase, and N-acetyllactosaminide beta-1,3-Nacetylglucosaminyltransferase were identified as a part of panel 1; while peptide belonging to GTPase IMAP family member 6 was a part of panel 2. Consecutive application of these two panels in an independent test set (n = 48 PC, n = 11 chronic prostatitis, n = 12 BPH, n = 4 healthy controls) resulted in AUC of 0.75 (*p* = 0.0001) [44].

#### 4.1.4. Tissue Biomarkers

In comparison to body fluids, tissue is the direct site of molecular alterations implicated in cancer onset and progression. Therefore, apart from the identification of proteins indicative of a disease state, tissue proteomics may allow for better understanding of disease biology, thus may guide identification of drug targets. However, high heterogeneity of tissue sample, i.e., by presence of other than cancerous cells, intratumor variability, etc., has to be taken into account when performing such type of analysis, as it may affect the subsequent interpretation of the results. Moreover, collection of tissue samples is invasive and associated with side effects. Several tissue proteomics studies have been performed in the context of PC biomarker development, as presented in the following section, with most of the studies targeting at the identification of biomarkers for risk stratification to guide intervention.

A. Biomarkers for risk stratification to guide therapeutic intervention

In two studies, focus was placed on the analysis of *N*-glycoproteins, as being selectively present in plasma [59,86]. In the first study, N-linked glycopeptides were investigated in tumor tissue samples from nonaggressive (n = 4), aggressive PC (n = 4), respective adjacent normal tissue, and normal prostate tissue from healthy transplant donors by using LC-MS/MS [86]. Comparative analysis of the relative glycoprotein abundance between aggressive vs. other types of samples was conducted, and 17 glycoproteins were shortlisted based on at least 50% change in the abundance in all comparisons. Among those, five proteins were significantly altered between aggressive and nonaggressive PC [86]. Differential expression of three of these, namely cartilage Oligomeric matrix protein, Periostin, and Membrane primary amine oxidase was validated using ELISA in an independent set of tissue samples (12 normal prostate tissue from transplant donors, 27 nonaggressive prostate tumor, 20 aggressive prostate tumors, and 10 prostate metastases) [86]. However, the performance of these proteins to discriminate between aggressive and nonaggressive PC warrants further investigation. In the second study, N-linked glycopeptides from normal prostate (n = 10), nonaggressive (n = 24), aggressive (n = 16), and metastatic (n = 25) tumor tissue samples were analyzed using sequential window acquisition of all theoretical fragment ion spectra mass spectrometry (SWATH MS), followed by a search against reference spectral library containing known *N*-glycosylation sites [59]. A total of 220 significantly altered glycoproteins were identified across the groups, including 50 glycoproteins significantly changed between aggressive and nonaggressive PC. Two, N-acylethanolamine acid amidase (NAAA) and PTK7 were selected for further validation based on the following criteria: (a) the prominent alteration between aggressive and nonaggressive PC, (b) the positive staining in Human Protein Atlas in PC tissue, (c) novelty, and (d) the prediction of signal peptide for secretion. Initial IHC staining was performed on six PC tissue samples and adjacent normal epithelium and was further expanded by including additional 336 tissue cores (224 tumor tissue and 112 adjacent normal tissue) [59]. NAAA was significantly decreased and PTK7 was significantly increased in aggressive PC (GS ≥ 4 + 3), compared to nonaggressive PC (GS6, GS3 + 4). A combination of these two proteins resulted in AUC of 0.80, which outperformed each marker alone [59].

Iglesias-Gato et al. combined Super-Stable Isotope Labeling by/with Amino acids in Cell culture (SILAC) and LC-MS/MS analysis to investigate the PC tissue proteome in 36 tissue samples (28 prostate tumors, eight adjacent nonmalignant tissues), aiming at identification of prognostic biomarkers for PC aggressiveness to improve stratification of patients for active surveillance or active treatment [60]. 649 differentially expressed proteins (FDR < 0.1) were identified between cancer and normal tissue. Further comparison between proteomes from aggressive (GS ≥ 4 + 3, GS8-9, n = 16) and nonaggressive PC (GS6 and GS3 + 4, n = 12) allowed for the identification of 127 differentially expressed proteins (*p* < 0.05) [60]. Among those, Proneuropeptide-Y (pro-NPY), the top upregulated protein in aggressive PC, was selected for further validation in two independent sets of tissue samples using IHC (a total of 752 cases) [60]. In both cohorts the level of pro-NPY alone, or together with the transcriptional regulator ERG (ERG), was predictive of PC mortality in patients with low grade PC. For low risk patients with high level of pro-NPY and positive ERG, a hazard ratio of 17.3 was reported (*p* < 0.0001, n = 147) [60]. Moreover, the mature form of NPY was also proposed as a plasma biomarker diagnostic for PC [79].

An eight-biomarker panel based on quantitative multiplex proteomics imaging was established to stratify patients for active surveillance or active treatment [24]. The panel was derived from 11 previously identified biomarkers [87]. Different combinations of markers were tested in a set of 381 PC patients to develop a model best correlating biopsy-based prognosis with radical prostatectomy results [24]. The final model comprised of Cullin-2, Derlin-1, RNA-binding protein FUS, Stress-70 protein, mitochondrial, Decaprenyl-diphosphate synthase subunit 2, phospho-S6-Ser235/236, Mothers against decapentaplegic homolog 4, and Nuclease-sensitive element-binding protein 1 was validated in an independent blinded study of 276 PC patients [24]. Clinical validation showed an AUC value of 0.68 (*p* < 0.0001, n = 274) to discriminate between “favorable pathology” (i.e., surgical/RP Gleason ≤ 3 + 4 and organ confined (≤T2)) vs. ”non-favorable” (i.e., surgical Gleason ≥ 4 + 3 or non-organ confined (T3a, T3b, N, or M) and an AUC value of 0.65 (p < 0.0001, n = 276) for discrimination between “GS6” (surgical/RP Gleason = 3 + 3 and localized ≤ T3a) vs. “non-GS6” (surgical Gleason ≥ 3+4 or nonlocalized (T3b, N, or M)) [24]. The above biomarker panel is currently commercially available as ProMark (as presented in Table 1). However, given the moderate performance in a moderate size cohort, further assessment of the value of these biomarkers in treatment decision making would be highly beneficial to support clinical implementation.

Kuruma et al. explored the value and biological relevance of Staphylococcal nuclease domain-containing protein 1 (SND1) as biomarker for PC [88]. SND1 was identified in a previous proteomics investigation, as being upregulated in androgen-independent vs. androgen-dependent cancers [89]. Based on IHC, gradual increase in SND1 expression from normal (n = 62), benign (n = 51), HGPIN (n = 42), and cancer (n = 62) was reported (*p* < 0.0001) [88]. The expression level of SND1 was also significantly correlated with Gleason score (*p* = 0.025). However, based on the follow-up data for 60 out of 62 patients, multivariate analysis did not confirm SND1 as an independent predictor for biochemical failure after RP (*p* = 0.21) [88].

Barboro et al. characterized Heterogeneous nuclear ribonucleoprotein K (hnRNP K) expression in the context of PC [90]. IHC analysis revealed that nuclear and cytoplasmic levels of hnRNP K were significantly higher in cancerous vs. normal tissue (*p* < 0.0001) [90]. Further assessment of nuclear matrix level of hnRNP K using WB showed an increase in PC vs. non-tumor tissues, and the level of hnRNP K was also significantly correlated with GS (*p* = 0.008). Importantly, higher nuclear matrix expression was associated with poor prognosis (n = 47, *p* = 0.032, hazard ratio = 2.95) [90]. hnRNP K could thus support stratification of patients into different prognostic subgroups.

Additional potential biomarkers have been initially identified in cell lines, followed by validation in tissue samples [91,92]. Glen et al. applied 8-plex iTRAQ to investigate the proteome of two isogenic panels of prostate cancer cell lines (LNCaP cell line panel/androgen responsive and PC3 panel/androgen insensitive), with different growth and metastatic characteristics [91]. Differential expression was assessed separately for each panel of cell lines, with parental cell lines considered as reference. Seventeen and eighteen proteins were found significantly altered with fold change of at least 1.5 in the LNCaP and PC3 panels, respectively [91]. Among these, Protein disulfide-isomerase A6 precursor (ERp5) was supported by ample evidence implicating this protein in PC progression, thus ERp5 was further validated using IHC (n = 130). The expression of ERp5 was significantly higher in premalignant lesions vs nonmalignant epithelium as well as in high Gleason grade (4–5) vs. low grade (2–3) [91]. Saraon et al. applied SILAC to investigate proteome changes associated with the development of CRPC. Comparison of LNCaP and its androgen independent derivative resulted in the identification of 88 differentially regulated proteins [92]. This included multiple proteins involved in ketogenesis pathway (3-hydroxy-3-methylglutaryl-CoA synthase 2, D-beta-hydroxybutyrate dehydrogenase, 3-hydroxymethyl-3-methylglutaryl-CoA lyase, succinyl-CoA:3-ketoacid-coenzyme A transferase 1 (ACAT1))—all upregulated in the androgen independent cell line. Further validation using IHC (n = 48) confirmed the higher abundance of these proteins in high-grade PC, with the most prominent changes for ACAT1 [92]. Moreover, overexpression of ACAT1 in castration-resistant metastatic lesions (i.e., lung and liver, n = 19, *p* = 0.0329; lymph node, n = 28, *p* = 0.0121; bone, n = 74, *p* = 0.0001) vs. normal tissue (n = 8) was detected [92].

B. Biomarkers for detection of cancer

Patients’ acceptance of tissue-based diagnostic tests might be problematic, mostly due to its invasive character. Therefore, the investigations are focused towards developing noninvasive diagnostic biomarkers in PC, as supported through numerous studies published in this area (see above); whereas only few and preliminary studies devoted to tissue-based diagnostic markers have been conducted so far, as outlined below.

Ummanni et al. analyzed 23 tissue samples from patients with BPH (n = 11) and PC (n = 12) using 2DE in combination with MS for subsequent protein identification [93]. A total of 88 differentially altered protein spots were found in PC vs. BPH (*p* < 0.05). Differential expression of Prohibitin in PC vs. BPH was confirmed by IHC (n = 13 BPH, n = 5 prostate intra-epithelial neoplasia, n = 18 PC) [93], but its diagnostic performance was not assessed.

In another study, Sun et al. identified Periostin as potential PC biomarker using iTRAQ-based quantification and LC-MS/MS [94]. IHC analysis showed that Periostin exhibited significantly higher expression in stroma of PC (n = 20) vs. BPH (n = 20), further supporting the proteomics findings [94].

MALDI-MS tissue imaging of cancer, benign epithelium and stromal areas of 13 RP specimens resulted in the identification of discriminatory peptide/ protein masses for cancer, benign and stromal tissue [95]. Analysis of additional 10 samples confirmed the highest discriminatory ability for peaks at m/z 6657.5 and 6284 for cancer tissue as well as a *m*/*z* 10775 peak for benign tissue [95]. Upregulation of Biliverdin reductase B (corresponding to m/z 6657.5) in PC vs BPH was supported by IHC (n = 376 including 94 benign and 282 malignant tissue cores) [95]. Further assessment of discriminatory potential of Biliverdin reductase B in a larger study is mandatory.

Jiang et al. focused on development of diagnostic biomarkers using proteomics and interactome analysis [96]. Sixty previously reported differentially expressed proteins, as identified by 2D-DIGE profiles of PC and adjacent benign tissue were studied. Protein–protein interaction analysis indicated 13 proteins with a high degree of connectivity, with three of them, i.e., PTEN, Histone deacetylase 1 (HDAC1) and Splicing factor, proline- and glutamine-rich (SFPQ) also closely interacting with each other [96]. The latter were validated using three publicly available transcriptomics datasets that were divided into training and test set. AUC values in a range of 0.89 and 0.93 were reported in the test data sets [96]. This preliminary result on the diagnostic performance needs to be further validated in independent and original study. IHC (n = 112 tumor and n = 29 adjacent benign prostate tissue) and ELISA (n = 22 tumor and n = 21 adjacent benign prostate tissue) analysis confirmed differential expression of PTEN, HDAC1, and SFPQ [96]. Moreover, applying multivariate analysis PTEN was identified as an independent prognostic marker for biochemical-recurrence free survival (p = 0.016) [96].

Similarly, Davalieva et al. applied 2D-DIGE in combination with MS to investigate differences in proteome from PC (n = 5) and BPH tissue (n = 5) [97]. Comparative analysis of protein patterns indicated significant changes in the abundance of 39 spots (fold ≥ 1.8), of which 28 distinct proteins were identified by MS. In silico Ingenuity analysis revealed existence of three functional networks, with Network 1 related to cell morphology, cellular assembly and organization, and cellular compromise, Network 2 related to cell cycle, connective tissue development and function, and organ morphology, and Network 3—cell cycle, organ morphology, and organismal development [97]. In addition, the Ingenuity search against disease patterns/ biomarkers related to PC and PC cell lines predicted 21 proteins as associated with PC. Three proteins involved in cell cycle regulation and progression (i.e., Ubiquitin-conjugating enzyme E2 N, Proteasome subunit beta type-6, and Serine/threonine-protein phosphatase PP1-beta catalytic subunit) were selected for validation using WB (n = 42), and differential expression was in line with proteomics analysis [97]. However, further assessment of the performance of these proteins in the specific clinical context is required to support their clinical applicability.

C. Biomarkers for detection of lymph node metastatic PC

Pang et al. investigated biomarkers associated with lymph node metastatic (LNM) PC. Biomarker discovery was performed in tissue samples (n = 10 patients with localized PC, n = 7 patients with lymph node metastatic PC, n = 10 BPH) using 2D-DIGE [69]. A total of 58 differentially expressed proteins between localized and metastatic PC were identified. Six proteins that have been related to metastases were further verified using WB (n = 9 localized PC, n = 9 LNM PC, n = 9 BPH) and IHC (n = 48 localized PC, n = 27 LNM PC, n = 30 BPH). Increase in the expression of FABP5, Methylcrotonoyl Coenzyme A carboxylase 2, Inorganic pyrophosphatase 2 isoform 1 precursor, Stomatin like protein 2, and Ezrin in metastatic PC patients was observed, while Transgelin was decreased in metastatic cancer vs. localized PC [69]. In addition, FABP5 was also evaluated in serum by ELISA (n = 30 BPH, n = 20 localized PC, n = 20 metastatic PC), further confirming the tissue-based findings [69].

## 5. Outlook

Advancements in the management of PC are urgently needed to improve on guiding biopsies and intervention and thus decrease disease burden. As presented above, proteomics has contributed to the identification of potential biomarkers for PC. Urinary and blood-based proteome have been extensively studied to identify noninvasive/minimally invasive biomarkers for cancer detection, while seminal plasma and tissue proteome was rather investigated as a source of biomarkers for risk stratification, in order to better discriminate between aggressive and nonaggressive disease phenotype. In any case, only a small number of investigations have been performed for identification of markers to predict treatment response.

As a result, a vast amount of proteomics data has been collected, a long list of molecular determinants of disease has been generated, and some of the shortlisted biomarker candidates could be verified in independent cohorts. In addition, initial assessment of their clinical utility showed rather good performance. This, in principle, confirms the potential value of proteomics findings. It is also becoming evident from multiple studies, that the combination of multiple markers into a panel increases the performance of the tests.

Although these results appear promising and the proteomics platforms are sufficiently advanced in analytical and technical terms, the identified biomarkers obviously have not reached the phase of clinical implementation. Based on the presented studies, some main factors can be listed accounting for this: (a) the study design is frequently not adjusted for the specific clinical context, (b) the investigations are not performed in the appropriate population, (c) the studies are underpowered, (d) comparison with the currently applied routine methodologies is missing, and (e) health economics evaluation for cost-effectiveness analysis is not always present.

In an effort to support implementation of biomarkers into practice (not only in the context of PC), several guidelines/recommendations have been established covering quality assessment, study design, reporting, and practical aspects [98,99,100,101,102,103]. Nevertheless, it seems that the gap between research activities and clinical application still exists. Since large amounts of proteomics data have been generated for PC and multiple proteins have been defined as potential biomarkers, we should now move towards testing of these markers in the specific clinical context in well-designed, appropriately powered prospective studies. This requirement has recently been discussed in detail for a similar disease: bladder cancer [104,105]. In parallel, studies evaluating the cost-effectiveness of the proposed markers and benefits over the available means should be assessed to ease the implementation process. Based on the results in the presented studies, development of biomarker panels seems to better address disease heterogeneity compared to single biomarkers. However, these efforts require a substantial amount of funding, along with extensive collaboration between clinicians, scientists, statisticians and stakeholders. This topic, as well as strategies on how to move towards implementation of proteomics have been recently discussed; with the main obstacles as well as possible solutions to have been outlined [106].

Collectively, a solid background has been established in the area of proteomics biomarkers for PC, aiming to address the main clinical needs. This holds a great promise to decrease overdiagnosis and overtreatment, to reduce the number of unnecessary/invasive biopsies, and also in the future to support selection of the best treatment option. Thus, it is now time to organize a way to bring these findings closer to clinical implementation.

## Figures and Tables

**Table 1:** Overview on the commercially available biomarker-based tests.

Applicability	Test	Biomarkers	Tissue Type	REF
**Who to biopsy?**	PSA *	PSA	Blood serum	[2]
	Prostate Health index *	Total, free, and p2PSA	Blood serum	[6]
	SelectMDx	2-gene panel: HOXC6 and DLX1	Urine after DRE	[7]
	ExoDx	3-exosome gene expression: PCA3, ERG, and SPDEF	Urine	[8]
	4Kscore Test *	4-protein panel: total PSA, fPSA, intact PSA, and human kallikrein 2	Blood plasma	[9]
	Mi-Prostate Score	2-gene panel: TMPRSS2:ERG and PCA3	Urine after DRE	[10]
**Who to re-biopsy?**	Progensa	lncRNA PCA3	Urine after DRE	[11]
	PSA *	PSA	Blood serum	[2]
	ConfirmMDx	3-gene panel (methylation status): GSTP1, APC, and RASSF	Biopsy	[12,13]
	4Kscore Test *	4-protein panel: total PSA, fPSA, intact PSA, and human kallikrein 2	Blood plasma	[14]
	Prostarix	4-metabolites panel: sarcosine, alanine, glycine, and glutamate	Urine after DRE	[15,16]
	Prostate Core Mitomic test	Quantification of a 3.4-kb mitochondrial DNA deletions	Biopsy	[17,18]
**Who to treat?**	OncotypeDX	17-gene panel: 12 cancer-related genes (AZGP1, KLK2, SRD5A2, FAM13C, FLNC, GSN, TPM2, GSTM2, TPX2, BGN, COL1A1, and SFRP4) and 5 housekeeping genes	Biopsy	[19,20,21]
	Prolaris	46-gene panel: 31 cell cycle progression and 15 housekeeping genes	Biopsy	[22,23]
	ProMark *	8-protein panel: DERL1, CUL2, SMAD4, PDSS2, HSPA9, FUS, pS6, and YBX1	Biopsy	[24]
**Who is likely to benefit from additional treatment?**	Decipher	22-gene panel: biomarkers involved in cell proliferation, cell differentiation, motility, immune modulation, and androgen receptor signaling	Radical prostatectomy	[25,26]
	Prolaris	46-gene panel: 31 cell cycle progression and 15 housekeeping genes	Radical prostatectomy	[27,28]
	AR-V7	AR-V7 mRNA status	Circulating tumor cells	[29,30,31,32]

Abbreviations: APC—Protein APC, AR-V7—Androgen-receptor splice variant 7 messenger RNA, AZGP1—Zinc-α2-glycoprotein, BGN—Biglycan, COL1A1—Collagen alpha-1(I) chain, CUL2—Cullin-2, DERL1—Derlin-1, DLX1—Homeobox protein DLX-1, DNA—Deoxyribonucleic acid, DRE—Digital rectal examination, ERG—Transcriptional regulator ERG, FAM13C—Protein FAM13C, FLNC—Filamin-C, fPSA—free PSA, FUS—RNA-binding protein FUS, GSN—Gelsolin, GSTM2—Glutathione S-transferase Mu 2, GSTP1—Glutathione S-transferase P, HOXC6—Homeobox protein Hox-C6, HSPA9—Stress-70 protein, mitochondrial, KLK2—Kallikrein-2, lncRNA—Long non-coding RNA, mRNA—messenger ribonucleic acid, p2PSA—[–2]proPSA, PCA3—Prostate Cancer gene 3, PDSS2—Decaprenyl-diphosphate synthase subunit 2, pS6—phospho-S6-Ser235/236, PSA—Prostate Specific Antigen, RASSF—Ras association domain-containing protein 1, SFRP4—Secreted frizzled-related protein 4, SMAD4—Mothers against decapentaplegic homolog 4, SPDEF—SAM pointed domain-containing Ets transcription factor, SRD5A2—3-oxo-5-alpha-steroid 4-dehydrogenase 2, TMPRSS2—Transmembrane serine protease 2, TPM2—Tropomyosin beta chain, TPX2—Targeting protein for Xklp2, YBX1—Nuclease-sensitive element-binding protein 1; * protein-based tests.

**Table 2:** Summary of clinical samples applied in prostate cancer biomarker research.

Sample Type	Advantages	Disadvantages	Applicability
Urine	− Easily obtainable − Noninvasive sampling − No side effects related to sampling − Low sampling cost − Available in large quantity − High proximity to tumor site	− No direct link to disease pathophysiology − Low protein concentration	− Cancer detection * − Guide biopsy − Risk stratification to guide intervention
Plasma/Serum	− Moderately invasive sampling − Low sampling cost − Available in high quantity	− High complexity − Broad range of protein concentrations − Distant from tumor site	− Cancer detection * − Risk stratification to guide intervention − Prediction of treatment response
Seminal plasma	− Minimally invasive sampling − Low sampling cost − Proximity to tumor site − High concentration of prostatic proteins	− Difficult collection in men with erectile dysfunction − High complexity − Broad range of protein concentrations	− Risk stratification to guide intervention * − Cancer detection
Tissue	− Site of cancer initiation and progression − Reflects disease underlying pathophysiology − Enables understanding of disease mechanisms	− Invasive sampling − Side effects of sampling collection may occur − High sampling cost − Restricted availability − Frequently limited quantity − Sampling error − High complexity	− Risk stratification to guide intervention * − Cancer detection − Detection of metastatic cancer

* most frequently studied application per sample type.

**Table 3:** Most promising proteomics-derived biomarkers for Prostate Cancer.

Biomarkers	Type of Sample ^†^	Sample Size ^†^	Methods	Performance ^†^	REF
**Detection of cancer**					
AZGP1	Urine after DRE	n = 127	WB	AUC = 0.68 (95% CI 0.59–0.78); **AZGP1 + PSA**: AUC = 0.75 (95% CI 0.66–0.85)	[55]
β2M + PGA3 + MUC3A	Urine	n = 173	WB	AUC = 0.71 (95% CI: 0.63–0.79); **Biomarkers + PSA categories**: AUC = 0.81 (95%CI 0.74–0.89)	[56]
TGM4 + ADSV	Urine after DRE/ EVs	n = 107	SRM	AUC = 0.65 (95%CI 0.55–0.76)	[57]
CE-MS biomarker panel	Urine	n = 213	CE-MS	AUC = 0.70; **Biomarkers + clinical variables**: AUC = 0.82 (95% CI/ not reported)	[45,47]
HYOU1 + ASPN + CTSD + OLFM4 + PSA	Serum	n = 105; Test set: n = 37	SRM	AUC = 0.84 (95% CI 0.82–0.96); Test set: Sensitivity of 64.3% sensitivity, 82.6% specificity	[58]
**Risk stratification to guide treatment**					
CUL2 + DERL1 + FUS + HSPA9 + PDSS2 + pS6+ SMAD4 + YBX1	Tissue	n = 276	QMPI	AUC = 0.68 (95% CI 0.61–0.74, N = 274) for “favorable pathology” vs. “non-favorable pathology” **^¥^** AUC = 0.65 (95%CI 0.58–0.72, *p* < 0.0001, n = 276) for “GS 6” vs. “non–GS 6” **^¥^**	[24]
NAAA + PTK7	Tissue	n = 336	IHC	AUC = 0.80 (95%CI 0.799–0.803)	[59]
pro-NPY + ERG	Tissue	n = 752	IHC	Level of pro-NPY alone or together with ERG is predictive of PC mortality in patients with low-grade PC	[60]

Abbreviations: ADSV—Adseverin, ASPN—Asporin, AUC—Area under the receiver operating characteristic curve, AZGP1—Zinc-α2-glycoprotein; β2M—Beta-2-microglobulin, CE-MS—Capillary Electrophoresis coupled to Mass Spectrometry, CI—Confidence Interval, CTSD—Cathepsin D, CUL2—Cullin-2, DERL1—Derlin-1, DRE—Digital Rectal Examination, ERG—Transcriptional regulator ERG, EVs—Extracellular vesicles, FUS—RNA-binding protein FUS, GS—Gleason Score, HSPA9—Stress-70 protein, mitochondrial, HYOU1—Hypoxia upregulated protein 1, IHC—Immunohistochemistry, MUC3A—Mucin-3A, NAAA—N-acylethanolamine acid amidase, OLFM4—Olfactomedin-4, PC—Prostate cancer, PDSS2—Decaprenyl-diphosphate synthase subunit 2, PGA3—Pepsin A-3, pro-NPY—Proneuropeptide-Y, pS6—phospho-S6-Ser235/236, PSA—Prostate-specific antigen, PTK7—Inactive tyrosine-protein kinase 7, QMPI—Quantitative multiplex proteomics imaging, SMAD4—Mothers against decapentaplegic homolog 4, SRM—Selected Reaction Monitoring, TGM4—Transglutaminase-4, WB—Western Blot, YBX1—Nuclease-sensitive element-binding protein 1. **^†^** Verification/Validation cohort, **^¥^** “favorable pathology”: Surgical Gleason ≤ 3 + 4 and organ confined (≤T2); “nonfavorable pathology”: surgical Gleason ≥ 4 + 3 or non-organ confined (T3a, T3b, N, or M); “GS 6”: Surgical Gleason = 3 + 3 and localized ≤ T3a; “non-GS 6”: surgical Gleason ≥ 3 + 4 or nonlocalized (T3b, N, or M).

## Supplementary Materials

The following are available online at http://www.mdpi.com/2075-4418/8/3/57/s1. Table S1. Complete list of the studies along with the abstracts retrieved through literature search using Web of Science. Search was performed on 6 June 2018. Articles that have passed the set citation threshold are highlighted in grey. Table S2. List of selected studies that have been presented in the context of this review. Table S3. List of promising biomarkers derived from proteomics investigations. Information on the purpose of the study, type of biological samples analyzed, cohort size, and biomarker performance are provided.

## Abbreviations

2D-DIGE—Two-dimensional difference gel electrophoresis, 2DE—Two-dimensional gel electrophoresis, ACAT1- Succinyl-CoA:3-ketoacid-coenzyme A transferase 1, ADSV—Adseverin, ALDH1A3—Aldehyde dehydrogenase 1A3, AMBP—Protein AMBP, AR—Androgen receptor, AR-V7—Androgen-receptor splice variant 7 messenger RNA, AUC—Area under the receiver operating characteristic curve, AZGP1—Zinc-α2-glycoprotein, BPH—Benign prostatic hyperplasia, CD63—CD63 antigen, CE-MS—Capillary electrophoresis coupled to mass spectrometry, CLIA—Clinical Laboratory Improvement Amendments, DRE—Digital rectal examination, eEF1A1—Eukaryotic translation elongation factor 1 alpha 1, ELISA—Enzyme-linked immunosorbent assay, EPS—Expressed prostatic secretions, ERG—Transcriptional regulator ERG, ERp5—Protein disulfide-isomerase A6 precursor, ERSPC—European Randomized study of Screening for Prostate Cancer, EVs—Extracellular vesicles, FABP5—Fatty acid-binding protein 5, FDA—U.S. Food and Drug Administration, GGT1—Gamma glutamyltransferase 1, GS—Gleason Score, HDAC1—Histone deacetylase 1, HGPIN—High-grade prostatic intraepithelial neoplasia, hnRNP K—Heterogeneous nuclear ribonucleoprotein K, HP—Haptoglobin, HSP90α—Heat shock protein 90 alpha, IHC—Immunohistochemistry, IMPDH2—Inosine monophosphate dehydrogenase II, iTRAQ—Isobaric tags for relative and absolute quantitation, LC-MS/MS—Liquid chromatography coupled with tandem mass spectrometry, LNM PC—Lymph node metastatic PC, MALDI-TOF/TOF—Matrix assisted laser desorption ionization combined with tandem time of flight MS, mCRPC—Metastatic castration resistance PC, MIC-1—Macrophage inhibitory cytokine 1, MRM—Multiple reaction monitoring, MS—Mass Spectrometry, MS/MS—Tandem mass spectrometry, MUC3A—Mucin-3A, NAAA—N-acylethanolamine acid amidase, NPY—Neuropeptide-Y, PARK7—Protein/nucleic acid deglycase DJ-1, PC—Prostate Cancer, PCA3—Prostate Cancer Gene 3, PEDF—Pigment epithelium-derived factor, PGA3—Pepsin A-3, pro-NPY—Proneuropeptide-Y, PROS1—Protein S, PSA—Prostate Specific Antigen, PTEN—Phosphatase and tensin homolog, PTK7—Inactive tyrosine-protein kinase 7, ROC—Receiver operating characteristic curve, RP—Radical prostatectomy, SAA1—Serum amyloid A protein, SDS—Sodium dodecyl sulfate, SFPQ—Splicing factor, proline—and glutamine-rich, SILAC—Stable Isotope Labeling by/with Amino acids in Cell culture, SND1—Staphylococcal nuclease domain-containing protein 1, SPON2—Spondin 2, SRM—Selected reaction monitoring, SWATH MS—Sequential window acquisition of all theoretical fragment ion spectra mass spectrometry, TF—Transferrin, TGM4—Transglutaminase-4, TSR1—pre-rRNA-processing protein TSR1 homologue, WB—Western Blot, β2M—β-2-microglobulin,
